# Gene Expression in Amnion-Derived Cells Cultured on Recombinant Laminin 332—A Preliminary Study

**DOI:** 10.3389/fmed.2021.719899

**Published:** 2021-11-10

**Authors:** Katarzyna Skowron-Kandzia, Marcin Tomsia, Halina Koryciak-Komarska, Danuta Plewka, Patrycja Wieczorek, Piotr Czekaj

**Affiliations:** ^1^Students Scientific Society, Faculty of Medical Sciences in Katowice, Medical University of Silesia, Katowice, Poland; ^2^Department of Cytophysiology, Chair of Histology and Embryology, Faculty of Medical Sciences in Katowice, Medical University of Silesia, Katowice, Poland

**Keywords:** human amniotic cells, extracellular matrix, basement membrane, laminin 332, integrins, gene expression, pluripotency, *in vitro* cell differentiation

## Abstract

Human amniotic cells (hAC) exhibit characteristics of undifferentiated cells and immunomodulatory properties. Recognition of the relationship between amniotic cells and components of the extracellular matrix is an important condition for their *ex vivo* preparation and further successful clinical application in regenerative medicine and transplantology. Laminin 332 (LN-332), as a natural component of the basement membrane of amniotic epithelial cells and a ligand for integrin receptors, may strongly influence the phenotype and fate of amniotic cells. We investigated the impact of recombinant LN-332 on hAC viability and expression of markers for pluripotency, early differentiation, adhesion, and immunomodulatory properties. During 14 days of culture, hAC were quantified and qualified by light microscopy, immunohistochemistry, immunocytochemistry, and flow cytometry. Gene expression was assessed with real-time polymerase chain reaction (RT-PCR) arrays and compared with differentiated cells originated from the three germ layers. LN-332 caused an over 2-fold increase in the total number of hAC, accompanied by a 75% reduction of SSEA-4-positive cells and an increase in HLA-ABC-positive cells. In particular, we observed that the presence of laminin 332 in the medium of a short-time culture modifies the effect of culture duration on hAC, enhancing time-dependent inhibition of expression of certain genes, including pluripotency and differentiation markers, laminin 332 subunits (which may be part of self-regulation of LN-332 synthesis by amniotic cells), and integrins. The changes observed in hAC were more distinct with respect to differentiated mesenchymal cells, resulting in more comparable phenotypes than those represented by differentiated endo- and ectodermal cells. We concluded that laminin 332 present in the culture medium influences to a certain extent proliferation, adhesion, and differentiation of amniotic cells in culture.

## Introduction

Human amniotic membrane constitutes a promising source of cells for regenerative medicine and transplantology. The amnion consists of two types of cells: epiblast-derived human amniotic epithelial cells (hAEC) lying on a thick basement membrane and human amniotic membrane-mesenchymal stromal cells (hAM-MSC) situated in an avascular stroma (1). Human amniotic cells (hAC) exhibit features of pluripotent stem cells, such as expression of pluripotency markers, e.g., octamer-binding transcription factor 4/POU class 5 homeobox1 OCT4/POU5F1, sex determining region Y—box 2 SOX2, Kruppel-like factor 4 KLF4, stage-specific embryonic antigens SSEA-4 and SSEA-3 and tumor-related antigens TRA1-60 and TRA1-81, as well as the ability to differentiate into cells representing all three germ layers (2–4). Unlike human embryonic stem cells (hESC), hAC are characterized by low telomerase activity, which is associated with weak tumorigenic activity, and lack or moderate expression of major histocompatibility complex (MHC) class II and I antigens, respectively, resulting in low cell immunogenicity (5, 6). They synthesize immunosuppressive compounds, e.g., HLA-G, and exhibit immunomodulatory properties (7).

The hAC phenotype is partially dependent on signaling molecules, including laminins, which are insoluble components of the extracellular matrix (ECM) (8). Laminins are heterotrimeric glycoproteins typically found in the basement membrane of epithelial cells. Several types of laminins have been recognized in the human placenta, namely: LN-211 (α2β1γ1), LN-221 (α2β2γ1), LN-332 (α3β3γ2), LN-311 (α3β1γ1), LN-321 (α3β2γ1), LN-511 (α5β1γ1), and LN-521 (α5β2γ1) (8, 9).

Laminins bind to cell membranes through integrin receptors, which are dimeric, transmembrane proteins consisting of various α (ITGA) and β (ITGB) chains. Among 24 different combinations of integrin subunits, four can interact with laminins: α3β1, α6β1, α6β4, and α7β1. Amniotic cells express mainly α3, α5, α6, β1, and β4 subunits (10). Integrins are responsible for signal transduction from the microenvironment into the cell. Laminin–integrin interactions trigger signaling pathways involved in cell survival and self-renewal. They contribute to cell adhesion, motility, proliferation, or differentiation (11).

Laminin 332 (LN-332) plays a substantial role in stem cell phenotyping. As a natural component of the hAEC basement membrane, it may strongly influence the fate of amniotic cells. LN-332 has the highest affinity for α6β4 integrin, which stabilizes the binding of cells to the basement membrane through hemidesmosomes (12). Moreover, LN-332 promotes cell polarization, proliferation, and apoptosis (13). A soluble form of LN-332 causes activation of protein kinase C (PKC), phosphatidyl inositol 3-kinase (PI3K), and mitogen-activated protein kinase (MEK) signaling pathways (14).

Appropriate xeno-free and chemically defined culture conditions would enable effective propagation or differentiation of amniotic cells. To date, most *in vitro* experiments determining the impact of recombinant laminins on cultured cells have been carried out on hESC, induced pluripotent stem cells (iPSC), or mouse embryonic stem cells (mESC) (15, 16). Little is known about phenotypic changes of hAC cultured *in vitro* in LN-332-coated flasks. Earlier studies determining the impact of LN-332 present in the cell culture medium have proved its pro-proliferative and pro-adhesive effect on human mesenchymal stem cells (hMSC) (17). Although LN-332 was shown to stimulate mESC proliferation, it also decreased the pluripotency of these cells (18).

Recognition of the relationship between amniotic cells and components of the ECM is an important condition for their *ex vivo* preparation and further successful clinical application. Thus, the aim of the study was to investigate the influence of recombinant LN-332 on the proliferative potential and viability of hAC and, especially, expression of major histocompatibility complex antigens and genes of pluripotency, laminins, integrins, integrin-dependent signaling pathways, and cell differentiation.

## Materials and Methods

### Human Placentas

Term placentas from 10 healthy donors aged 27–35 years were obtained from the Department of Gynecology and Obstetrics of the District Railway Hospital in Katowice. The placentas were collected after uncomplicated elective cesarean deliveries performed at 37–39 weeks of gestation for medical reasons, namely previous cesarean section, tokophobia, transverse position of the fetus, or placenta previa. If no evidence of placental insufficiency was found, the placentas were promptly transferred to the laboratory in phosphate-buffered saline (PBS) (Sigma) containing an antibiotic-antimycotic solution [0.1 U/ml penicillin, 0.1 mg/ml streptomycin sulfate, 0.25 μg/ml amphotericin B (PAA)] and 5 mM EDTA (Invitrogen), then rinsed several times to remove blood.

Human placentas were collected with the consent of the Bioethical Committee of the Medical University of Silesia in Katowice (Decision No. KNW/0022/KB/228-1/14).

### *In vivo* Immunodetection of Integrin Subunits in the Human Amnion

To confirm the presence of integrin subunits *in vivo*, the amniotic membrane was manually separated from the chorion, washed in PBS supplemented with antibiotics and antimycotics, and cut into small pieces (20 × 20 mm). The fragments destined for ITGA6 and ITGB4 immunodetection were fixed in 4% buffered formalin (pH 7.4) for 3 h at room temperature (RT) and infiltrated with 30% sucrose for cryoprotection (in PBS overnight at 4°C). The samples were then embedded in tissue-freezing medium (OCT Embedding Matrix, CellPath), frozen, and cut with microtome (CRYOCUT, American Optical Corp.) into 7 μm slices.

For immunodetection of integrin subunits ITGA6 and ITGB4, the sections were washed twice with PBS, incubated in 0.2% Triton X solution for 15 min, blocked with 1% bovine serum albumin (BSA) for 1 h (RT), and incubated overnight with a specific primary antibodies (Table 1) dissolved at 1:100 or 1:50. Afterwards, the samples were incubated with 0.2% Triton X solution for 30 min, and then with fluorochrome-conjugated secondary antibodies (Table 1). To visualize cell nuclei, the sections were mounted in HardSet Mounting Medium with DAPI (Vector). In control samples, primary antibodies were substituted with isotype-specific immunoglobulins (Table 1).

**Table 1:** Antibodies and isotype controls used for immunodetection of integrin subunits and CK-7.

**Antigen**	**Primary antibody**	**Secondary antibody**	**Isotype control**
ITGA6	Anti-integrin alpha 6 antibody, clone MP 4F10 (Abcam)	TRITC Goat anti-Mouse IgG H&L (Abcam)	Purified Mouse IgG2b, κ Isotype Control (BioLegend)
ITGB4	Anti-integrin beta 4 antibody, clone 439-9B (Novus Biologicals)	Alexa Fluor 488 Mouse monoclonal Anti-Rat IgG2b heavy chain (Abcam)	Rat IgG2b Isotype Control (Novus Biologicals)
ITGB1	Anti-integrin beta 1 antibody, clone EP1041Y (Abcam)	Anti-Rabbit IgG, MP-7401 (Vector) + DAB	Purified Rabbit IgG Isotype Control (Antibodies-online)
CK7	Anti-cytokeratin 7 antibody, clone OV-TL 12/30 (Abcam)	Goat anti-Mouse IgG DyLight 488 (Abcam)	Mouse IgG1 kappa monoclonal, clone MOPC21 (Abcam)

The fragments designed for ITGB1 immunodetection were fixed in 4% buffered formalin (1 h, RT), dehydrated, embedded in paraffin blocks, and then cut into 5 μm slices (microtome HM 350S, Zeiss). The slices were next dewaxed and rehydrated with xylene and alcohol series. Antigen unmasking was carried out in a water bath (30 min., 95°C, citrate buffer, pH 6.0). After cooling, the slices were washed with distilled water and PBS with 0.05% Tween 20 (PBS-T). Then incubation in 3% H_2_O_2_ for 10 min was carried out to inhibit endogenous peroxidase activity. The samples were blocked (2.5% horse serum, 30 min.) and incubated overnight with a primary antibody at a concentration of 0.45 μg/ml (dilution 1:400), or an appropriate isotype control (Table 1). Then, ImmpPRESS Reagent, Anti Rabbit IgG, MP-7401 (Vector) was used for 30 min, followed by its substrate diaminobenzidine (DAB), according to the manufacturers' protocols.

### Cultures of Differentiated Cells

As additional controls for the experiment, short-time cultures were performed of differentiated human cells originating from the three germ layers. Normal human epithelial keratinocytes (NHEK), human small airway epithelial cells (HSAEC), and human aortic endothelial cells (HAEC) (all from Lonza) were thawed and cultured in dedicated media (KGM Gold Keratinocyte Growth Medium BulletKit, SAGM Small Airway Epithelial Cell Growth Medium, and EBM-2 Endothelial Basal Medium-2, respectively).

### Isolation and Culture of hAC

The amniotic membrane was manually separated from the chorion, washed several times in PBS containing antibiotics and antimycotic agents, and cut into small pieces (15 × 15 mm). The fragments were then subjected to enzymatic digestion with 2.4 U/ml dispase (Roche) for 7 min, 0.05% trypsin (PAA) twice for 40 min, and 0.75 mg/ml collagenase (Roche) for 60 min at 37°C. The cells released after each digestion were collected by centrifugation (300 g, 5 min., 4°C), suspended in a culture medium (TeSR2 Kit; StemCell Technologies), depleted of red blood cells by incubation with lysing buffer (Becton Dickinson), and counted with an automated cell counter (MOXI Z Mini; Orflo). Then, hAC were divided into two groups: cultured in recombinant LN-332-coated flasks (25 cm^2^; Sarstedt) and uncoated (control) flasks (1 × 10^5^ cells/cm^2^) in TeSR2 medium in the incubator and collected after 12, 36, and 72 h as well as 7 and 14 days. In order to coat the flask with LN-332, the subunits α3, β3, and γ2 (Biolamina) were diluted in 1 x DPBS (Ca^2+^, Mg^2+^) to a concentration of 5 μg/ml and left in the flasks overnight at 4°C. The cells were passaged after reaching 80% confluence (three passages were performed during 14 days of cell culture). The culture medium was changed every 48 h. Cell number and viability were assessed at each time point (12, 36, 72 h, 7 days, and 14 days) with a MOXI Z automated cell counter (Orflo) and with the MTT.

### *In vitro* Immunodetection of the CK7 Epithelial Marker

For preliminary characterization of hAC population, detection of the epithelial cell marker CK7 was performed with an indirect immunofluorescence technique. After 12 h of cell culture, non-adherent and adherent cells were centrifuged together (300 g, 5 min.) and suspended at a concentration of 1 × 10^7^ cells/ml in PBS, fixed, and permeabilized in ice-cold methanol (10 min, −20°C). Next, the cells were blocked with a mixture of 1% BSA, 10% normal goat serum (NGS), and 0.3 M glycine in 0.1% PBS-Tween (1 h, RT) and then incubated (1 h, 4°C) with a specific primary antibody, diluted 1:40, and incubated (1 h, RT in the dark) with a secondary antibody conjugated with fluorochrome. Simultaneously, an isotype control was prepared (Table 1). All antibodies were used in accordance with the manufacturer's protocol. The cells were fixed in VECTASHIELD Hard-set Mounting Medium with DAPI (Vector).

### Microscopic Examination

All the images taken from cell cultures were analyzed using a Nikon Eclipse Ti-U fluorescence microscope equipped with a Nikon Digital Sight DS-SMc camera running NIS-Elements AR 3.00 software (Nikon Instruments Inc.) or, in the case of immunohistochemical staining, using a Nikon Eclipse E600 microscope equipped with a Sony SSC-DC58AP camera.

### Flow Cytometry

In order to obtain qualitative and quantitative analyses, cell surface markers, namely pluripotency marker SSEA-4, major histocompatibility complex antigens (HLA class I, HLA class II, HLA-G), and integrin subunits: ITGA6, ITGB1 and ITGB4 were detected by flow cytometry. Cells obtained from the amniotic membrane were harvested sequentially after 12, 36, 72 h, 7 days, and 14 days of cell culture in TeSR2 medium in uncoated or LN-332-coated flasks. Adherent and non-adherent cells were collected, suspended in a staining medium (PBS containing 10% FBS and 5 mM EDTA), and incubated (1 × 10^6^ cells) for 30 min (4°C, in the dark) with 5–20 μl of specific antibody solution or appropriate fluorochrome-conjugated isotype control, according to the manufacturer's protocols (Table 2). Cytometric analyses were performed using the FACS Aria flow cytometer (Becton Dickinson) with the FACSDiVa software.

**Table 2:** Antibodies and isotype controls used for flow cytometry.

**Antigen**	**Specific antibody**	**Isotype control**
SSEA-4	Mouse Anti-Human/Mouse SSEA-4 PerCP-conjugated Monoclonal Antibody (R&D Systems)	PerCP Mouse IgG3, Isotype Control (R&D Systems)
ITGA6	FITC Rat Anti-Human CD49f (BD Pharmingen)	FITC Rat IgG2a, κ Isotype Control (BD Pharmingen)
ITGB1	APC Mouse Anti-Human CD29 (BD Pharmingen)	APC Mouse IgG1, κ Isotype Control (BD Pharmingen)
ITGB4	PE Rat Anti-Human CD104 (BD Pharmingen)	PE Rat IgG2b, κ Isotype Control(BD Pharmingen)
HLA-ABC	APC Mouse Anti-Human HLA ABC (BD Pharmingen)	APC Mouse IgG1, κ Isotype Control (BD Pharmingen)
HLA-DR,DP,DQ	FITC Mouse Anti-Human HLA DR, DP, DQ (BD Pharmingen)	FITC Mouse IgG2a, κ Isotype Control (BD Pharmingen)
HLA-G	PE Mouse Anti-Human HLA G (Abcam)	PE Mouse IgG1 Isotype Control (BD Pharmingen)

### Real-Time Polymerase Chain Reaction (RT-PCR) Microarray Assay

Transcriptome analysis was performed to compare the gene expression profile of hAC cultured on recombinant LN-332 with control gene expression of both hAC cultured in uncoated flasks and differentiated cells. RNA was isolated from the cells (2.5 million per sample) using the RNeasy Mini Kit (Qiagen). Total RNA quality and quantity were determined by measuring the absorbance spectra in a NanoDrop 2,000 UV/Vis spectrophotometer (ThermoScientific). cDNA was generated and amplified from 740 ng RNA isolated from each sample using the RT2 First Strand Kit (SA Biosciences). The RT-PCR reaction was performed on custom-made 96-well plates with RT^2^ SYBR Green qPCR Mastermix kit and Custom RT^2^ Profiler PCR Array system (Qiagen).

Each plate contained primers for 27 genes of interest, appropriate RNA quality controls, and two housekeeping genes. The genes of interest represented five groups: (1) transcription factors responsible for cell stemness: *NANOG, POU5F1, SOX2, KLF4, and MYC*; (2) laminin and integrin subunits: *LAMA1, LAMA3, LAMA5, LAMB1, LAMB3, LAMC1, LAMC2, ITGA6, ITGB1, and ITGB4*; (3) markers of differentiation toward the three germ layers: ectoderm (*EN2)*, endoderm (*HNF4A)*, and mesoderm (*DES)*; (4) markers of early differentiation/progenitor cells: mesenchymal stem cells (MSC), namely *CD44, CD73 (NT5E), CD90 (THY1), CD105(ENG);* neurons, namely *NES;* hepatocytes, namely *FOXA2 (HNF3B);* and cardiomyocytes, namely *HAND1;* and (5) integrin-dependent signaling pathways (cell survival, adhesion, and migration): *PTK2 (FAK)* and *PIK3R5*. Amplification was performed with Roche Light Cycler 480. The detection threshold was set to at least 2-fold.

### Statistical Analysis

Statistical analysis was performed with the use of Statistica 8.0 software. A Chi-square test was performed and the data were analyzed with the standard Mann–Whitney *U* test, Kruskal–Wallis test, ANOVA test, and Student's *t*-test, as appropriate. Gene expression analysis was carried out using the manufacturer's software (RT^2^ Profiler PCR Array Data Analysis Template v3.5; https://geneglobe.qiagen.com/pl/; SA Biosciences, QIAGEN). The significance level was set at *p* < 0.05.

## Results

### *In vivo* Immunodetection of Integrin Subunits in the Human Amnion

**Figure 1:**
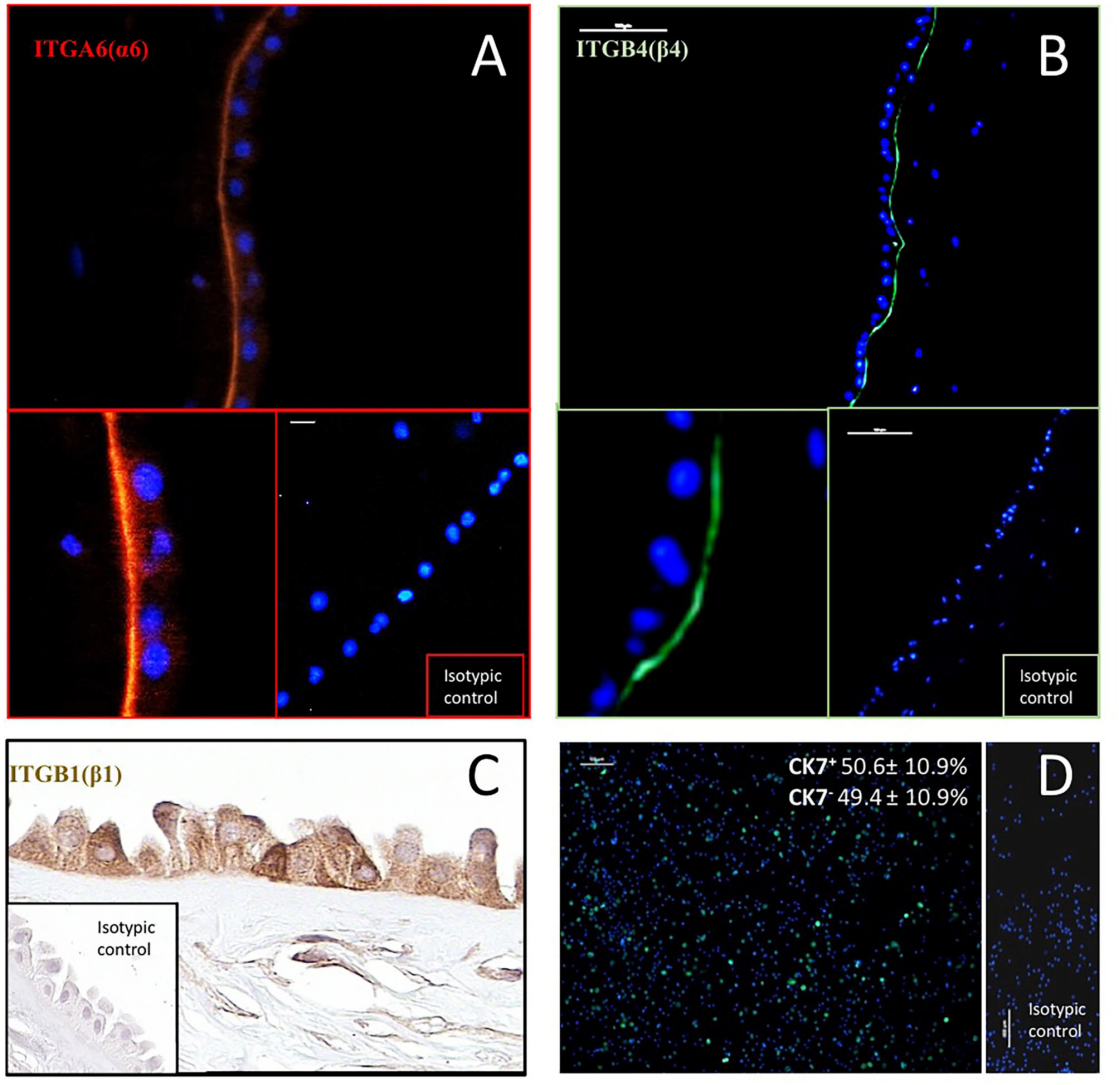
Immunodetection of integrin subunits: ITGA6(α6) **(A)**, ITGB1(β1) **(C)** and ITGB4(β4) **(B)** in human amnion, and cytokeratin 7 (CK7)–a marker of epithelial cells–in hAC after 12 h of culture **(D)**. In immunofluorescence **(A,B)**, antibodies were conjugated with TRIC (orange) and Alexa Fluor 488 (green). Anti-CK7 antibody was conjugated with DyLight 480 (green). Cell nuclei were stained with DAPI (blue). Percentage of CK7^+^ and CK7^−^ hAC cells (mean ± SD) is also given (*n* = 10). ITGB1 was detected by immunohistochemical ABC method. Magnification: ITGA6–200*x*, and 400*x*, scale 10 μm; ITGB4–100*x*, and 400*x*, scale 100 μm; ITGB1–400*x*; CK-7–100*x*, scale 100 μm.

The potential ability of hAC to interact with LN-332 present in their microenvironment was confirmed by immunodetection of integrin receptor subunits. ITGA6 (α6) and ITGB4 (β4) were detected in the basal part of the hAEC plasma membrane. Integrin ITGB1 (β1) was present in both the basolateral and apical plasma membranes. However, a positive reaction was also seen in the cytoplasm of hAEC and in the cytoplasm and plasma membrane of hAM-MSC (Figure 1).

### Efficiency of hAC Isolation

The efficiency of hAC isolation from the amniotic membrane of the examined placentas was, on average, 129.5 million cells (±38.48), and the number of hAC obtained varied from 98 to 204 million (median: 121 million). An average number of 18.65 ± 6.06 million isolated hAC per gram of amniotic tissue was counted. This number varied from 10.1 to 27.6 million/g (median 16.7 million/g) and was not related to the weight of individual amniotic membranes, which ranged from 5.29 to 11.94 g (median: 6.46; mean: 7.40 ± 2.37).

### *In vitro* Immunodetection of the Epithelial Cell Marker CK7

Epithelial cells were identified in the primary culture using an anti-CK7 antibody. The average CK7^+^/hAC ratio was 50.6% (Figure 1), but individual differences were observed in hAEC rates from 34.3 to 62.4% (not shown).

### Characteristics of hAC in an *in vitro* Culture

**Figure 2:**
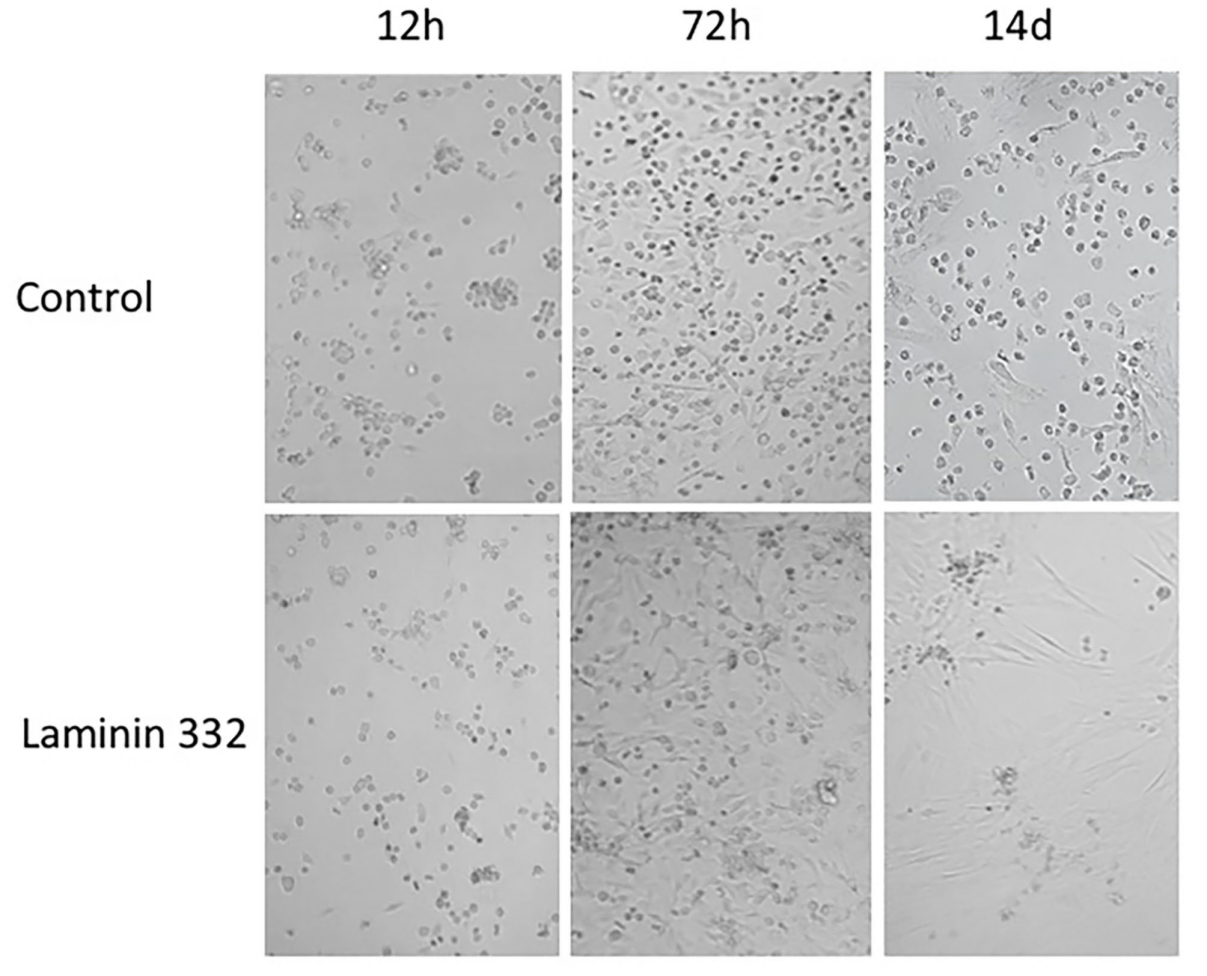
Morphology of hAC cells cultured 14 days on uncoated and laminin-coated plates. Different proportions of adherent and non-adherent cells after 12, 72 h, and 14 days of culture can be seen (Magn. 100*x*).

During cell culture, morphological differences were observed between cells cultured on recombinant LN-332 and in uncoated flasks. Cells cultured on LN-332 exhibited stronger attachment to the surface of the culture flask and formed longer protrusions (Figure 2).

**Figure 3:**
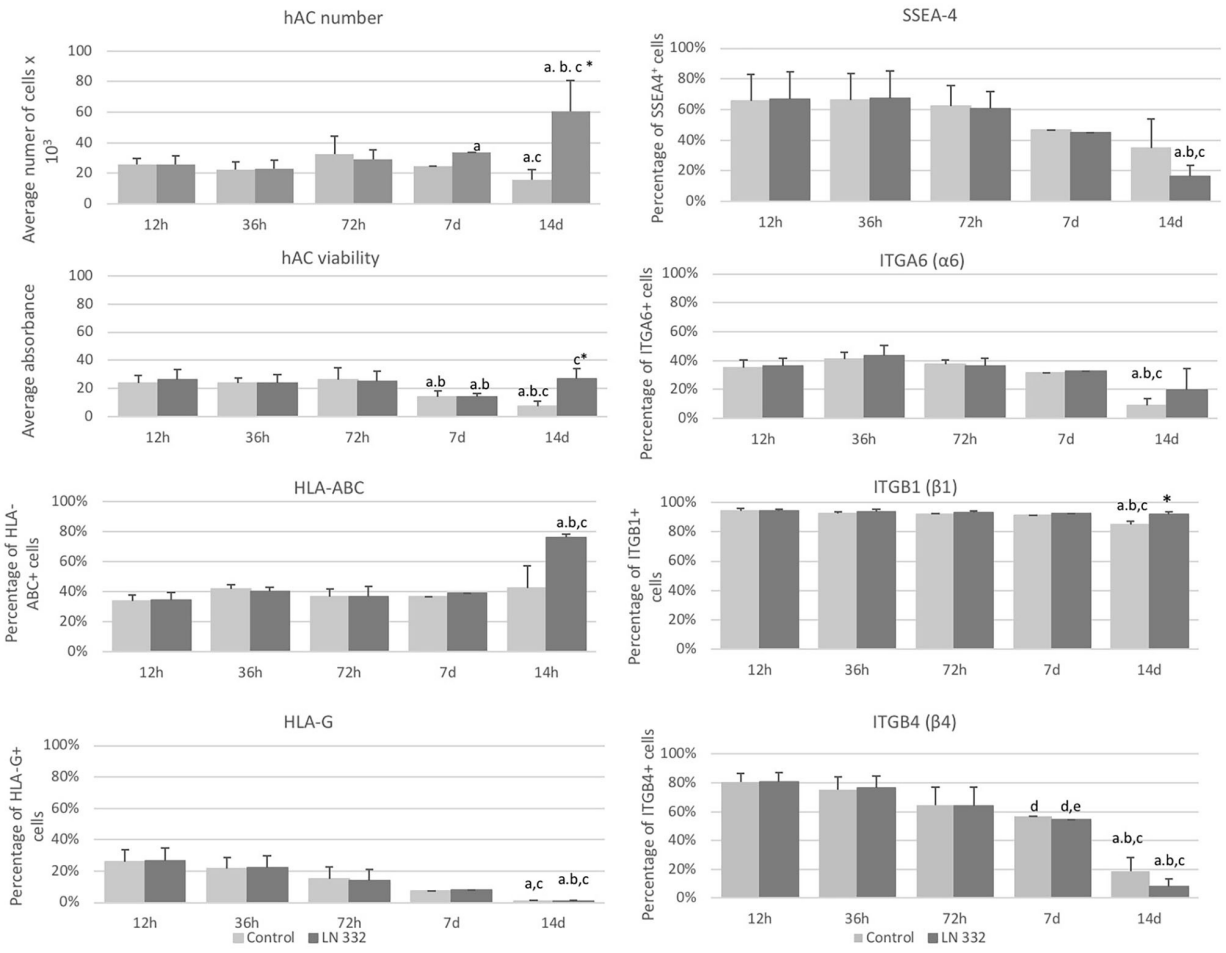
Average number and viability of amniotic cells cultured on uncoated (control) and laminin 332-coated (LN-332) plates. Differences statistically significant as compared to: ^*^appropriate control; ^a^12 and 36 h; ^b^72 h; ^c^7 days; *p* < 0.05; *n* = 5. Number of SSEA-4^+^, HLA-ABC^+^, HLA-G^+^, ITGA6^+^, ITGB1^+^, and ITGB4^+^ cells among hAC cultured on uncoated (Control) and laminin 332 (LN-332) coated plates. Differences statistically significant as compared to: ^*^ appropriate control ^a^12 h, 36 h, and 7 d; ^b^72 h; ^c^12 h; ^d^36h; *p* < 0.05.

The number of cells cultured in uncoated flasks decreased significantly (*p* < 0.05) on day 14. On the other hand, from day seven of culture, we observed a statistically significant (*p* < 0.05), over 2-fold increase in number of cells cultured on LN-332 compared to both earlier time points and appropriate controls (Figure 3).

The viability of hAC cultured in uncoated flasks diminished significantly from day seven of culture. The decrease of hAC viability in laminin-coated flasks observed on day 7 was temporary and did not occur on day 14 (Figure 3).

### Surface Marker Expression on Isolated hAC

At the beginning of the cell culture (12 h), population of SSEA4^+^ hAC was about 66% and diminished 4-fold on day 14 (*p* < 0.05) in the cells cultured in LN-332-coated flasks (Figure 3).

Populations of ITGB1^+^ cells (94%) and ITGB4^+^ cells (80%) were much more numerous than of ITGA6^+^ cells (35%). The numbers of ITGA6^+^ and ITGB1^+^ cells were stable throughout 7 days of culture and decreased in uncoated flasks on day 14 by 74 and 10% (*p* < 0.05), respectively (Figure 3). The number of ITGB4^+^ cells decreased significantly: on day 7 of culture by 30% in both investigated groups and on day 14 by 90.3 and 76.5% in LN-332-coated and uncoated flasks, respectively (Figure 3). The differences in ITG expression between the control and experimental groups were very small or statistically insignificant (Figure 3).

Simultaneously with the decreasing number of SSEA4^+^, ITGB1^+^, and ITGB4^+^ cells, we observed a significant increase in the number of SSEA4^−^/ITGA6^−^, SSEA4^−^/ITGB4^−^, and SSEA4^−^/ITGB1^+^ cells on day 14. The changes in the co-expression of SSEA-4 and ITGs were more advanced in hAC cultured in LN-332-coated flasks compared to the control medium (Table 3).

**Table 3:** Percentage of hAC exhibiting co-expression of pluripotency marker SSEA-4 with integrins or HLA antigens among amnion cells cultured on uncoated (Control) and LN332-coated plates.

**Phenotype**		**Control**					**Laminin 332**				
		**12 h**	**36 h**	**72 h**	**7d**	**14 d**	**12 h**	**36 h**	**72 h**	**7 d**	**14 d**
**SSEA-4+**	ITGA6+	26.5	32.5	29.6	18.4	**7.9** **^c^**	28	34.8	29.7	19.1	**6.4** **^b^ ^c^ ^d^**
	ITGB1+	65	66.3	62	45.3	33.7	66.8	67.3	50.8	45.0b	**16.7** **^a^ ^b^ ^c^ ^d^**
	ITGB4+	59.7	58.8	50.6	27.9	**17.0** **^b^ ^c^ ^d^**	61.1	60	52.4	27.6	**6.9** **^a^ ^b^ ^c^ ^d^**
	HLA-ABC+	18.8	26.7	20.6	17.4	8	19.1	25.6	18.9	18	8.6
	HLA-G+	20	17.2	11.7	4.3	**0.9** **^d^**	20.5	17.5	11.3	4.9	**0.6** **^d^**
	ITGA6-	39.6	34.1	33.8	30	26.8	40	33.3	33.5	28.6	**10.9** **^a^ ^b^ ^c^ ^d^**
	ITGB1-	0	0	0.2	**1.2** **^b^ ^c^**	0.5	0	0	0	0.7^c^	0.4
	ITGB4-	5	7.4	11.4	**18.5** **^a^ ^b^**	17	5.4	6.8	11.1	**18.0** **^a^ ^b^**	9.5
	HLA-ABC-	48.6	40.8	46	**30.5** **^c^**	28.2	48	42.8	43	29	**9.7** **^a^ ^b^ ^c^ ^d^**
	HLA-G-	47.2	50.3	51.8	43.5	35.2	47.2	50.8	50.6	42.1	**17.6** **^c^ ^d^**
**SSEA-4-**	ITGA6+	8	7.2	7.2	12.6	1	7.8	8.2	7.3	12.9	12.7
	ITGB1+	29.8	27.1	31.3	47.8	55.7	28.4	27.1	33.4	49.5	**79.0** **^a^ ^b^ ^c^ ^d^**
	ITGB4+	20.9	16.6	14.2	28.8	**2.2** **^d^**	19.7	16.6	14.2	26.7	**1.5** **^d^**
	HLA-ABC+	14.6	15.4	16.1	22.5	36.4	14.9	14.3	18.2	22.3	**70.1** ^*^ ^a^ ^b^ ^c^ ^d^
	HLA-G+	5.9	4.3	3.5	3.1	0	5.9	4.6	2.9	3.4	**0.1** **^d^**
	ITGA6-	25.9	26.1	29.4	39	64.3	24.2	23.7	30.5	39.4	**70.1** **^a^ ^b^ ^c^**
	ITGB1-	5.2	6.5	6.5	**5.7** **^c^**	**10.0** **^a^ ^b^ ^c^ ^d^**	4.8	5.6	5.7	4.8	3.9^*^
	ITGB4-	14.4	17.2	23.8	24.8	**63.9** **^a^ ^b^**	13.8	16.6	22.3	**27.7** **^a^**	**82.1** **^a^ ^b^ ^c^ ^d^**
	HLA-ABC-	18	17.1	17.3	**29.6** **^c^**	27.3	17.2	17.3	19	30.7	**11.6** ^*^ ^d^
	HLA-G-	26.8	28.2	32.9	49.1	**63.9** **^a^**	26.4	27	35.2	49.6	**81.6** **^a^ ^b^ ^c^ ^d^**

*Differences statistically significant comparing to*:

a *12 h*,

b *36 h*,

c *72 h*,

d *7 days*;

* *control*;

*p < 0.05. All statistically significant differences are written in bold*.

The percentage of HLA-ABC^+^ cells remained at a level of 35–40% and increased to 75.8% on day 14 of culture on LN-332 (Figure 3). Thus, as the number of SSEA-4^+^ cells decreased, there was a significant (*p* < 0.05) increase (from 14.9 to 70.1%) of SSEA4^−^/HLA-ABC^+^ cells (Table 3). On the other hand, 12 h after cell isolation, the HLA-G antigen was present on the surface of about 26% hAC, regardless of the applied culture medium. During cell culture, we observed a decrease in the number of HLA-G^+^ cells in both LN-332-coated and uncoated flasks (Figure 3). This was associated with a statistically significant increase (to 81% in laminin-coated flasks and to 64% in the control) of SSEA4^−^/HLA-G^−^ cells (Table 3).

By contrast, only a very small number (< 1%) of hAC expressing HLA class II was noted. There was no change in the number of cells expressing HLA II during cell culture (not shown).

### Gene Expression

**Figure 4:**
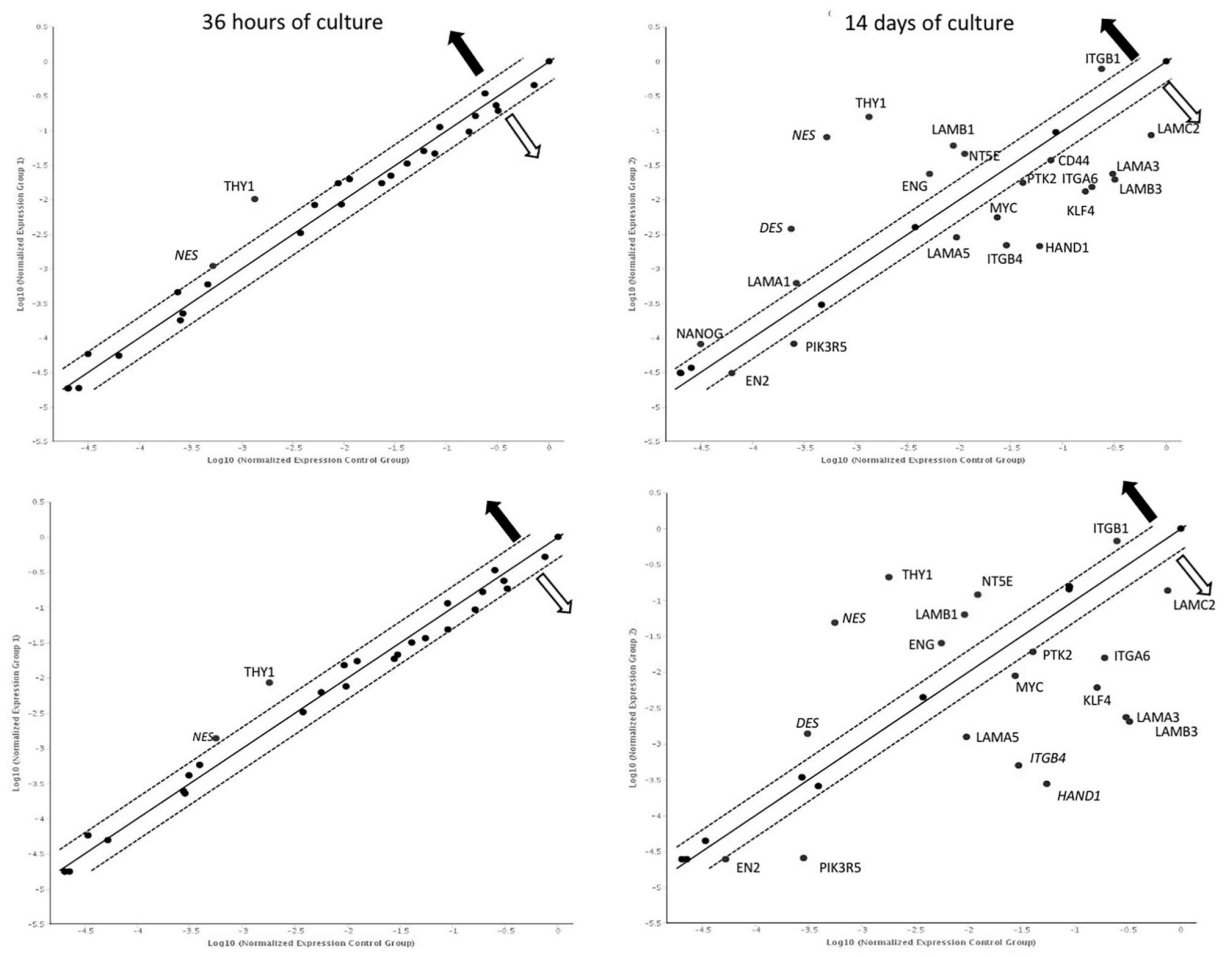
Gene expression profile after 36 h and 14 days of culture on uncoated (up) laminin 332-coated (down) plates comparing to 12 h. Dotted line: expression changed 2-fold (statistically significant; *p* < 0.05); 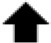
—overexpression 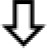
—underexpression.

After 36 h of culture, overexpression of *NES* and *THY1* genes was observed in both cultures compared to 12 h. After 14 days, significantly more genes with changed expression were observed in both the control and LN-332-coated flasks (22 and 19 genes, respectively; Figure 4).

**Figure 5:**
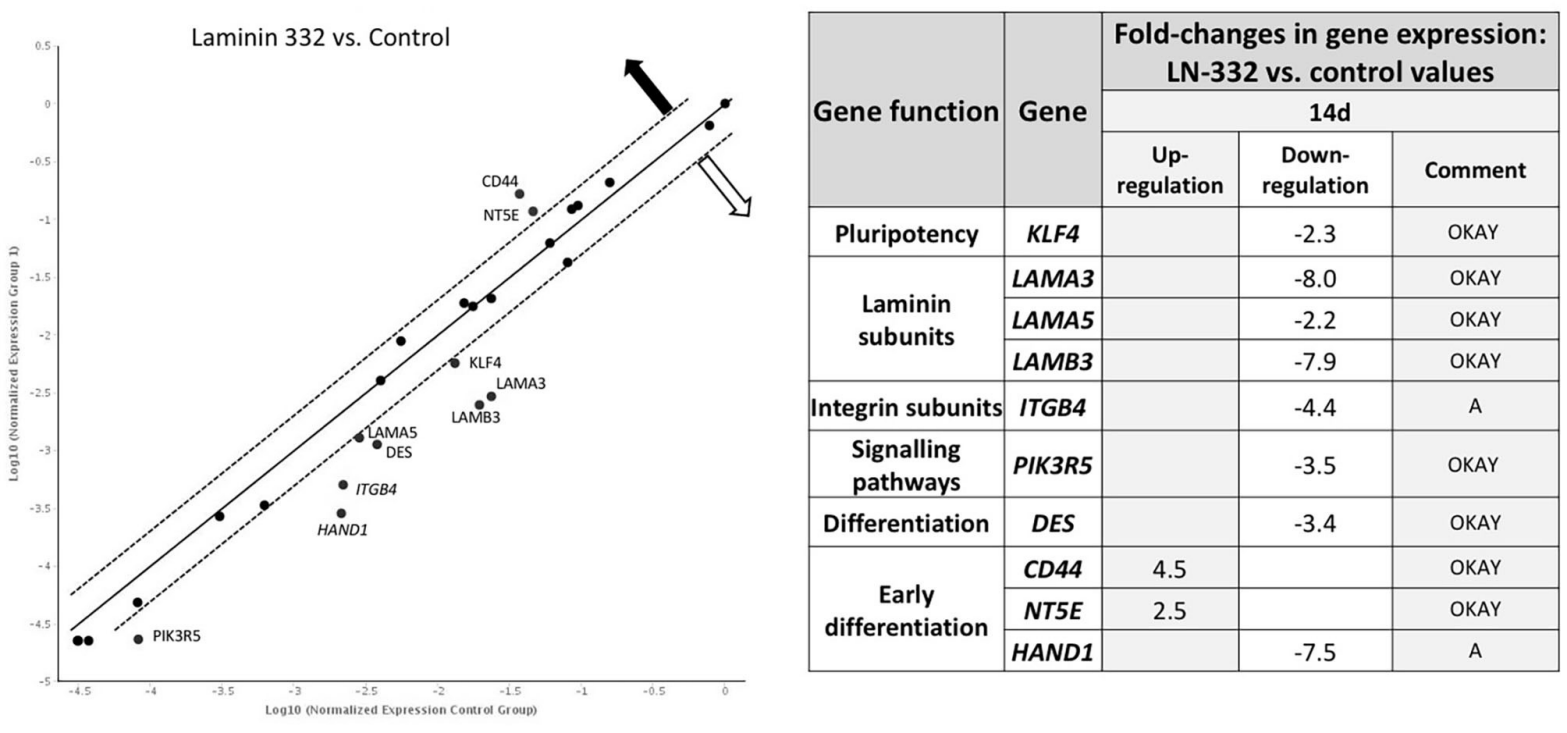
Gene expression profile after 14 days of culture on LN332-coated plates comparing to control plates. Table shows statistcally significant fold-changes in gene expression. Dotted line: expression changed 2-fold (*p* < 0.05). OKAY: The gene's expression is relatively high in both the test and control group. The threshold cycle (CT) is <30. A, This gene's average threshold cycle is relatively high (>30) in one sample and is reasonably low in the other sample (<30). These data mean that the gene's expression is relatively low in one sample and reasonably detected in the other sample; 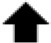
—overexpression 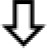
—underexpression.

A comparison of gene expression between hAC cultured in the two media revealed differences only on day 14 of the experiment. We noted two overexpressed genes (*CD44, NT5E*) and eight down-regulated genes (*KLF4, LAMA3, LAMA5, LAMB3, ITGB4, PIK3R5, DES*, and *HAND1*) in hAC cultured on LN-332 compared to the control (Figure 5).

Time-dependent changes in gene expression were mainly observed after 14 days of culture in all groups of the investigated genes. Among the pluripotency genes, the strongest down-regulation was observed for the *KLF4* gene (12.4 and 28.6 under-expression in the cells cultured in control and laminin-coated flasks, respectively). The *MYC* gene was also down-regulated, but to a lesser extent (−4.2*x* and −3.1*x*, respectively). The *NANOG* gene was slightly up-regulated in control flasks (Table 4).

**Table 4:** Gene expression fold-change after 14 days of culture on control and LN-332-coated plates comparing to 12 h of culture.

**Gene function**	**Gene name**	**Control**	**Laminin 332**
Pluripotency	*NANOG*	2.6	0
	*KLF4*	−12.6	−28.6
	*MYC*	−4.2	−3.1
Laminin subunits	*LAMA1*	2.3	2.4
	*LAMA3*	−12.9	−104.1
	*LAMA5*	−3.3	−7.5
	*LAMB1*	6.9	6.7
	*LAMB3*	−16.3	−133.1
	*LAMC2*	−8.3	−6.2
Integrin subunits	*ITGA6*	−12.5	−10.2
	*ITGB1*	3.3	2.6
	*ITGB4*	−13	−59.2
Integrin-dependent kinases	*PTK2*	−2.3	−2.3
	*PIK3R5*	−3	−12.2
Differentiation	*EN2*	−2	−2.3
	*DES*	16^A^	3.7^A^
Early differentiation	*CD44*	−2.1	0
	*NT5E*	4.1	9.5
	*THY1*	118.2	116
	*ENG*	4.6	3.7
	*NES*	154^A^	75.8^A^
	*HAND1*	−28	−192^A^

*The values are fold-changes higher or lower than 2, which was set as minimal statistically significant (p < 0.05). A, This gene's average threshold cycle is relatively high (> 30) in either the control or the test sample and is reasonably low in the other sample (< 30). These data mean that the gene's expression is relatively low in one sample and reasonably detected in the other sample. Other values—the gene's expression is relatively high in both the test and control group. The threshold cycle (CT) is <30*.

Laminin genes showed different levels of expression. *LAMA1* and *LAMB1* genes were up-regulated (Table 4). Most of the examined genes were down-regulated in both media. In the case of *LAMA3, LAMA5*, and *LAMB3* genes, we observed significantly stronger down-regulation in laminin-coated flasks. Both *LAMA3* and *LAMB3* genes were down-regulated about eight times compared to the control (Figure 5). Down-regulation of the *LAMC2* gene was comparable in both media (Table 4).

Genes encoding integrin subunits *ITGA6* and *ITGB4* were down-regulated more strongly in LN-332-coated flasks. In contrast, the expression of *ITGB1* gene was up-regulated on both media to a similar extent (3.3*x* and 2.6*x* in control and LN-332-coated flasks, respectively). The expression of both examined integrin-dependent kinases was down-regulated. Only the PIK3R5 gene exhibited significant differences between control and laminin-coated flasks (−3.5*x* down-regulation; Figure 5, Table 4).

We observed significant changes in the expression of genes responsible for differentiation, namely a slight under-expression of the *EN2* gene (ectoderm), similar on both media, and an up-regulation of the *DES* gene (mesoderm), which was significantly stronger in the control medium (16*x*) compared to LN-332-coated flask (3.7*x*; Table 4).

Early differentiation genes were mostly up-regulated. We noted a minor down-regulation of the *CD44* gene in the control medium and significant under-expression of the *HAND1* gene (characteristic for cardiomyocytes) in the control medium (28*x*) and in LN-332-coated flasks (192*x*).

The most pronounced changes were in mesenchymal differentiation genes, namely *NT5E, THY1, ENG*, and *NES* (Table 4). Statistically significant differences between the culture media were noted in the case of the *NT5E* gene (2.5 times stronger expression on LN-332; Figure 5); however, expression of the *NES* gene was stronger in the control medium.

During 14 days of culture, differences were observed between amniotic and differentiated cells originating from the ectoderm and endoderm. In contrast, a comparison between amniotic and mesenchymal cells revealed differences diminishing over time. These relationships were distinctly noticeable in the case of *LAMA3, LAMB3, LAMC2, ITGA6, ITGB4, NES*, and *ENG* genes (Supplementary Table 1).

## Discussion

In this study, we applied enzymatic digestion with dispase, trypsin, and collagenase to isolate hAC (both hAEC and hAM-MSC) from the amniotic membrane. Over half of hAC were epithelial cells, what means that the population of interest was heterogenous and more representative of the composition of the human amniotic tissue *in vivo*. It is known that hAEC derived from different amniotic membrane regions vary in their proliferative capacity and the expression of the pluripotency markers (19). We did not observe any relationships between the number of isolated cells, amniotic membrane weight, and weight of the placenta, which indicates individual differences in the cellular composition of the amnion (4, 20, 21).

The effect of enhanced adhesion of hAC was predictable as the recombinant fragment E8 of laminin 332 proved to increase adhesion of hESC and iPSC more than Matrigel or the entire LN-332 molecule. Interestingly, application of integrin α6β1 blocking antibodies attenuated cellular adhesion, indicating that LN-332-integrin α6β1 complex plays a pivotal role in cell–ECM interaction (22).

It is known that hAC do not proliferate in their natural environment and remain quiescent in pregnancy at term (23). However, freshly isolated hAEC entered the cell cycle and were able to form a confluent monolayer of cells in the culture (23). Moreover, it has been proved that co-culture of both epithelial and mesenchymal amniotic stem cells results in better proliferation activity and survival rate of hAM-MSC (24). The *in vitro* proliferative capacity of hAC is strongly dependent on the cell culture medium. For example, hAEC failed to grow in serum-free media: Dulbecco's Modified Eagle Medium (DMEM), Cn T22, and StemPro MSC but expanded until passage 5 in the EpiLife culture medium supplemented with S7 additive (25). Also, vitamin C was reported to promote hAC proliferation (17). Usually, DMEM supplemented with 10% FBS and 10 ng/ml of epidermal growth factor (EGF) is used (26), but certain medium modifications can stimulate hAC differentiation into cells of different germ layers (27). We used TeSR2, a serum-free medium dedicated to stem cells and free of complementary growth factors, such as EGF. After 14 days of observation, we noted a 40% decrease in the number of hAC cultured in uncoated flasks and 2-fold increase in the number of cells cultured on LN-332. Other authors reported a significant reduction in the proliferative potential of native hAC after the fifth passage (20, 28). The regulation of cell proliferation seems to be closely related to the relationships between ECM components and integrins (29). The enhanced proliferation of mESC strongly correlated with their adhesion when they were cultured on LN-332 and LN-511 (18). The data indicate that interaction of LN-332 with ITGA3, ITGA6, and ITGB1 subunits is responsible for the proliferative activity of ESC, MSC, and hAC (30). Harvesting a high number of cells from xeno-free cell culture system seems to be especially important regarding hAC potential clinical application (31).

Expression of SSEA-4, a glycosphingolipid described for the first time in embryonal carcinoma, has been proved in ESC as well as in MSC derived from different tissues, i.e., umbilical cord blood or hAM-MSC (32). In our study, the percentage of isolated SSEA-4^+^ hAC was about 66%. In previously published reports, this value ranged from 30 to 95% (3, 20, 33). We also observed individual differences in the number of SSEA-4^+^ cells between placentas. The expression of some other pluripotency markers, such as Oct-4, as well as differentiation capacity of amniotic cells appear to be strongly dependent on the donor (20, 21). It has also been reported that hAC characteristics depend on the area of origin of the amniotic membrane and onset of labor (34, 35). In this study, such differences between isolated hAC were reduced because the cells were always isolated from the placental part of the amnia. During cell culture, we observed a clear downward trend in the number of control cells expressing SSEA-4, but it was not statistically significant. Other authors showed a stable SSEA-4 expression pattern throughout hAEC culture until passage 3 (21). On the other hand, Bilic et al. described reduced SSEA-4 expression during 90 days of hAC culture (20). Thus, the results are inconsistent and probably strongly dependent on the cell subpopulation of interest and culture conditions (culture medium, additional growth factors applied).

Experiments carried out on hESC have proved that recombinant LN-332 enhances proliferation of these cells while simultaneously preserving their pluripotency features, such as the expression of SSEA-4, Oct-4, TRA 1–60, and TRA 1–81, throughout 10 passages (36). We observed a significant decrease in the number of SSEA-4^+^ hAC cultured on LN-332. However, no changes were observed in the expression of pluripotency genes, such as *NANOG* or *POU5F*. Alterations in *MYC* gene expression were observed during cell culture but were not so advanced as in *KLF4* gene expression. A decrease in *KLF4* gene expression was significantly stronger in laminin-coated flasks. KLF4 factor is necessary for laminin α3A chain gene expression (37). Moreover, a decrease in *KLF4* expression causes cell differentiation and is required for the induction of epithelial-to-mesenchymal transition (EMT) (38, 39). As laminin 332 restricts differentiation potential of hAC, its application may enable deriving a more differentiated cell population. Nonetheless, a more detailed studies are needed in order to describe the direction of these changes.

The pluripotency characteristics associated with the expression of the integrin α6 subunit were shown in hMSC derived from umbilical cord blood. It was demonstrated that this expression is regulated by direct OCT4 and SOX2 binding to the ITGA6 gene promoter (11). We observed a significant decrease in ITGA6 expression in hAC cultured in uncoated flasks, which was associated with a decrease in SSEA-4 expression. A reduction of integrin ITGA6 expression during hAC culture has been reported previously and may indicate cell differentiation and/or EMT (25). In hAC, ITGA6 usually dimerizes with integrin ITGB4 within the hemidesmosomes. Such a dimer (α6β4) is a major receptor for LN-332, localized in the basal cell membrane of epithelial cells (12). In our study, ITGA6 expression was concomitant with ITGB4. However, a significant decrease in the latter expression was observed already on the seventh day of culture. Thus, a reduction of ITGA6^+^/ITGB4^+^ was simultaneous with a decrease in the SSEA-4^+^ cell number. Moreover, a reduction of *ITGA6* and *ITGB4* gene expression correlated with a reduction of *PTK2* and *PIK3R5* gene expression, representing integrin-dependent signaling pathways. *ITGB4* gene expression in hAC became similar to differentiated mesenchymal cells but not to respiratory epithelium or keratinocytes. It can be assumed that the *SNAIL* gene product may play a role in the observed changes, as it inhibits the expression of integrin *ITGA6* and *ITGB4* genes and takes part in EMT regulation in epithelial cells (40).

The third analyzed integrin subunit was ITGB1. Its expression did not alter during cell culture, which is consistent with a study carried out by Pratama et al. (25). The presence of recombinant LN-332 in the culture flask stabilizes the number of ITGB1^+^ cells and, to a lesser extent, the number of ITGA6^+^ cells, while maintaining the adhesive properties of hAC. It seems that ITGB1 plays a significant role in cell adhesion *in vitro* conditions due to its presence on most of the examined cells and its stable expression during cell culture.

We observed that during hAC culture, gene expression of laminins *LAMA3, LAMA5, LAMB3*, and *LAMC2* genes is distinctly reduced and expression of *LAMA1* and *LAMB1* genes is increased. It can potentially modulate cell adhesion and signal transduction through integrin receptors. Similar results were obtained *in vitro* studies on hESC (41). In turn, recombinant LN-332 inhibited the expression of two genes, *LAMA3* and *LAMB3* and, to a lesser extent, *LAMA5*. Comparable outcome was obtained for colorectal cancer cell lines, where the presence of LN-332 in the culture medium resulted in reduced expression of the *LAMA5* gene (19). We suppose that the intensity of laminin gene expression depends on the presence of this laminin in the culture medium. Recombinant LN-332 probably inhibits expression of its own genes (*LAMA3* and *LAMB3*) in a negative feedback loop. A role of KLF4 factor cannot be excluded (37). We also observed that lower *LAMA3* and *LAMB3* gene expression correlated with reduced *PI3KR5* gene expression. Other authors obtained corresponding results indicating a relationship between laminin 332 and PI3K kinase gene expression (42). Inhibition of the kinase resulted in lower laminin expression. Furthermore, recent studies on pancreatic ductal adenocarcinoma cells revealed that knockdown of individual LAM genes resulted in different cell activity (proliferation/migration/EMT) (13). It is known that soluble factors, such as TGF-β, EGF and PGGF play a role (43).

The low immunogenicity of amniotic cells seems to be essential for their future clinical applications. The unique immunological characteristics of hAC make them a promising source of stem cells for regenerative medicine and transplantology. Firstly, our study confirmed that the percentage of HLA-DR, DP, DQ-positive cells was lower than 1% (5, 7, 20, 44, 45), and did not change during cell culture. Secondly, we obtained 34.4% hAC immunopositive for HLA class I antigen (HLA-ABC) among cells cultured in uncoated flasks and their number increased significantly in cells cultured on LN-332. A similar increase was observed in a study on hAEC differentiation into pancreatic and hepatic cells (46). Moreover, the co-expression analysis revealed that the decrease in the SSEA-4^+^/HLA-ABC^−^ cell number was associated with increased SSEA-4^−^/HLA-ABC^+^ cell number. Thus, differentiation of hAC on LN-332 cannot be excluded, and when they lose pluripotency features, they simultaneously become more immunogenic.

In addition to their low immunogenicity, hAC are unique in their ability to modulate the immunological response in many ways (7, 47). The presence of HLA-G is more evident on cells derived from term amniotic membranes than on cells from early amnia (48). HLA-G plays multiple immunomodulatory roles (7, 49, 50). We found that only 26% of the isolated hAC are HLA-G positive cells and 76% of these cells are characterized by simultaneous expression of HLA-G and SSEA-4. Other authors obtained both lower (3.4 ± 3%) (51) and much higher (75.8%) numbers of HLA-G^+^ cells (3). The gradual decrease in HLA-G expression observed by us during the 14 days of cell culture indicates decreasing immunosuppressive properties of hAC in both laminin-coated and uncoated flasks, which is consistent with previous reports (25, 52). Thus, laminin 332 does not influence immunomodulatory hAC properties associated with HLA-G expression, but enhances immunogenicity as HLA-ABC is elevated.

Changes in the expression of early differentiation genes as well as pluripotency markers suggested a gradual loss of epithelial characteristics by hAC with simultaneous gaining of mesenchymal features (23). We noted the most evident alterations in the expressions of such genes as *DES*, a marker of mesenchymal differentiation and one of the earliest myogenesis markers (53), *NES*, characteristic for early mesenchymal differentiation but also for neuronal progenitor cells (54), *THY1* (CD90), expressed on the surface of MSC as well as in neuronal cells, fibroblasts, or activated endothelial cells (55), *NT5E* (CD73), described on MSC but also on the surface of human T and B lymphocytes, and *ENG* (CD105), playing a role in cell adhesion and migration, described on endothelial cells or the syncytiotrophoblast (56). In our experiment, most of the analyzed markers of mesenchymal cells (*NT5E, THY1*, and *ENG*) showed enhanced expression during hAC culture in uncoated flasks. Considering that the native population of cells was a mixture of both hAEC and hAM-MSC, it can be assumed that increased expression of *THY1, DES, NT5E, ENG*, and *NES* genes indirectly suggests an EMT transition occurring in hAC culture. We suppose that LN-332 present in the culture medium can inhibit that process. We observed a 3.4 times lower expression of the *DES* gene in cells cultured in laminin-coated flasks as well as a weaker increase of *NES* expression and slight increase of the *NT5E* gene on LN-332 compared to the controls. Our observations are based on a comparison of the control and LN-332 groups of hAC as well as cultured hAC and differentiated cells representing the three germ layers. These observations can also be confirmed by the fact that EMT was inhibited in mammary epithelial cells cultured on a laminin-rich medium (Matrigel) (57).

Currently, about 12 clinical trials is carried out using hAC (31), but further, more detailed preclinical studies, should improve our knowledge and give a more detailed understanding of laminin 332–amniotic cells interaction. The impact of laminin 332 on EMT process seems to be complex and obtained results would be enriched. Among others, an assessment of kinase's activity or transcriptional factor's nuclear/cytoplasmic translocation, double immunostaining enabling more precise description of cell phenotype alterations of hAEC and hAM-MSC subpopulations, final verification of CK7/E-cadherin/vimentin in immunofluorescence and/or flow cytometry giving insight into EMT process, and an evaluation of differentiation potential toward the lineages of the three germ layers based on functional properties.

To sum up, in this study we verified the impact of human recombinant LN-332 on the hAC phenotype during short-time culture. We observed that the presence of laminin 332 in the culture medium enhances proliferation and immunogenic properties of hAC and reduces expression of some genes of pluripotency and differentiation as well as genes of laminin 332 subunits, which may be part of self-regulation of LN-332 synthesis by amniotic cells (Table 5). The changes observed in hAC were more distinct with respect to differentiated mesenchymal cells, resulting in more comparable phenotypes than those represented by differentiated endo- and ectodermal cells.

**Table 5:** Summary of significant effects of LN-332 on proliferative activity and specific markers of pluripotency, adhesion, immunomodulation and differentiation.

**Characteristic**	**Molecule**	**Method**	**Time-dependent changes**	**Laminin 332 effect**
Proliferation		Cell counter	↑	↑
Adhesion		Light microscopy	↓	↑↑
	ITG A6	FC	↓	Stabilization
		RT-PCR	↓	↓
	ITG B1	FC	↓	Stabilization
		RT-PCR	↑	↑
	ITG B4	FC	↓	↓↓
		RT-PCR	↓	↓↓
	LAMA3	RT-PCR	↓	↓↓
	LAMA5	RT-PCR	↓	↓
	LAMB3	RT-PCR	↓	↓↓
Pluripotency	SSEA-4	FC	↓	↓↓
	KLF4	RT-PCR	↓	↓↓
Immunomodulation	HLA-ABC	FC	Stabilization	↑↑
	HLA-G	FC	↓	↓
Differentiation	DES	RT-PCR	↑↑	↑
	NES	RT-PCR	↑↑	↑

*FC, flow cytometry*.

## Data Availability Statement

The original contributions presented in the study are included in the article/Supplementary Files, further inquiries can be directed to the corresponding author.

## Ethics Statement

The studies involving human participants were reviewed and approved by The Bioethical Committee of the Medical University of Silesia in Katowice (Decision No. KNW/0022/KB/228-1/14). The patients/participants provided their written informed consent to participate in this study.

## Author Contributions

KS-K: designed the study, acquired, analyzed, and interpreted the data, wrote the article, and made critical revisions. MT: contributed to the study design, cell culture, flow cytometry, and microarray assays. HK-K: flow cytometry data analysis. DP: immunohistochemical studies. PW: cell culture, immunocytochemical studies, and flow cytometry. PC: designed the study, analyzed, interpreted the data, wrote the article, made critical revisions, and gave the final approval of the version to be published. All authors contributed to the article and approved the submitted version.

## Funding

This work was funded by Ministry of Science and Higher Education of Poland (Diamond Grant No. 0044/DIA/2014/43 received by KS-K).

## Conflict of Interest

The authors declare that the research was conducted in the absence of any commercial or financial relationships that could be construed as a potential conflict of interest.

## Publisher's Note

All claims expressed in this article are solely those of the authors and do not necessarily represent those of their affiliated organizations, or those of the publisher, the editors and the reviewers. Any product that may be evaluated in this article, or claim that may be made by its manufacturer, is not guaranteed or endorsed by the publisher.
