# Supplementary material for: Gene Expression in Amnion-Derived Cells Cultured on Recombinant Laminin 332—A Preliminary Study
Source: Front Med (Lausanne). 2021 Nov 10;8:719899. doi: 10.3389/fmed.2021.719899 (PMC8631290; doi:10.3389/fmed.2021.719899)
Supplement: Supplementary file 1 [file Table_1.DOCX]

**Supplementary Table 1**

Selected genes’ expression fold-change after 14 days of culture on control and LN-332-coated plates comparing to differentiated cells originating from three germ layers: airway epithelium (endoderm), keratinocytes (ectoderm) and endothelium (mesenchyme). After 14 days of culture a comparison between the expression of some genes of amniotic and mesenchymal cells revealed differences diminishing over time making the phenotype of these cells more comparable. The values are fold-changes higher or lower than 2 which was set as minimal statistically significant, p<0.05. A: This gene’s average threshold cycle is relatively high (> 30) in either the control or the test sample. and is reasonably low in the other sample (< 30). These data mean that the gene’s expression is relatively low in one sample and reasonably detected in the other sample.

| **Gene** | **Medium** | **Airway epithelium**  (Endoderm) | | | **Keratinocytes**  (Ectoderm) | | | **Endothelium** (Mesenchyme) | | |
| --- | --- | --- | --- | --- | --- | --- | --- | --- | --- | --- |
|  |  | 12h | 36h | 14d | 12h | 36h | 14d | 12h | 36h | 14d |
| **KLF4** | **Control** | 7.2 | 4.2 |  | 15.4 | 8.9 |  | 446.8^A^ | 259.9^A^ | 35.6^A^ |
|  | **LN-332** | 7.1 | 4.0 | -4.0 | 15.1 | 8.7 |  | 440.8^A^ | 252.5^A^ | 15.4^A^ |
| **LAMA3** | **Control** |  |  | -11.1 |  |  | -14.7 | 238.1 | 181.2 | 18.5 |
|  | **LN-332** |  |  | -89.4 |  |  | -118.3 | 239.7 | 187.0 |  |
| **LAMA5** | **Control** | -4.4 | -4.8 | -14.3 |  |  | -3.8 |  | -2.0 | -6.1 |
|  | **LN-332** | -4.2 | -5.4 | -31.7 |  |  | -8.5 |  | -2.3 | -13.5 |
| **LAMB3** | **Control** |  |  | -13.8 |  |  | -18.0 | 82.3 | 49.9 | 5.0 |
|  | **LN-332** |  |  | -108.7 |  |  | -142.1 | 85.2 | 47.8 |  |
| **LAMC2** | **Control** |  | -2.3 | -11.9 |  |  | -7.6 | 419.3 | 265.8 | 50.3 |
|  | **LN-332** |  |  | -8.4 |  |  | -5.4 | 442.0 | 306.6 | 71.6 |
| **ITGA6** | **Control** |  |  | -14.4 |  |  | -15.7 | 4.3 | 3.7 | -2.9 |
|  | **LN-332** |  |  | -11.7 |  |  | -12.7 | 4.3 | 3.7 | -2.4 |
| **ITGB4** | **Control** | -5.1 | -6.6 | -66.5 | -4.4 | -5.7 | -57.4 | 73.7^A^ | 57.4^A^ | 5.7^A^ |
|  | **LN-332** | -4.9 | -6.8 | -289.6^A^ | -4.2 | -5.9 | -249.8^A^ | 77.1^A^ | 55.3^A^ |  |
| **PIK3R5** | **Control** | 15.5 | 11.2 |  | 15.14 | 10.9 |  | 15.8 | 11.4 |  |
|  | **LN-332** | 17.6 | 14.2 |  | 17.1 | 13.9 |  | 17.9 | 14.5 |  |
| **DES** | **Control** | 14.6 | 28.4 | 235.0^A^ | 14.3 | 27.7 | 229.1^A^ | 14.9 | 28.9 | 238.9^A^ |
|  | **LN-332** | 19.2 | 25.7 | 69.9^A^ | 18.7 | 25.0 | 68.2^A^ | 19.5 | 26.1 | 71.0^A^ |
| **CD44** | **Control** | -5.5 | -9.1 | -11.3 | -3.6 | -5.9 | -7.4 |  |  | -2.5 |
|  | **LN-332** | -4.7 | -8.6 | -2.5 | -3.0 | -5.6 |  |  |  |  |
| **NT5E** | **Control** | -3.4 |  |  | -4.3 | -2.4 |  | -3.2 |  |  |
|  | **LN-332** | -3.1 | -2.2 | 3.1 | -3.9 | -2.8 | 2.4 | -3.0 | -2.1 | 3.2 |
| **ENG** | **Control** | 2.4 | 4.0 | 11.1 | 60.9^A^ | 99.5^A^ | 279.5^A^ | -50.7 | -31.1 | -11.1 |
|  | **LN-332** | 2.6 | 2.9 | 9.7 | 66.4^A^ | 73.9^A^ | 244.4^A^ | -46.5 | -41.8 | -12.6 |
| **NES** | **Control** | 3.8 | 8^A^ | 583.6^A^ | 12.1 | 25.6^A^ | 1870.1^A^ | -331.1^A^ | -156.9 | -2.2 |
|  | **LN-332** | 4.1 | 10.1^A^ | 307.3^A^ | 13.0 | 32.4^A^ | 984.6^A^ | -309.4^A^ | -124.0 | -4.1 |
| **HAND1** | **Control** | 3710.3^A^ | 3134.8^A^ | 132.3^A^ | 3617.2^A^ | 3056.2^A^ | 129.0^A^ | 3770.8^A^ | 3185.9^A^ | 134.5^A^ |
|  | **LN-332** | 3407.9^A^ | 2273.7^A^ | 17.7 | 3322.4^A^ | 2216.7^A^ | 17.3 | 3463.5^A^ | 2310.8^A^ | 18.0 |
